# Development of molecular diagnostic protocols for simultaneous identification of common bed bugs (*Cimex lectularius*) and tropical bed bugs (*Cimex hemipterus*)

**DOI:** 10.1186/s13071-024-06447-7

**Published:** 2024-10-14

**Authors:** Jeong Heum Han, Junhyeong Choi, Susie Cho, Si Hyeock Lee, Ju Hyeon Kim

**Affiliations:** 1 Department of Tropical Medicine and Parasitology, Seoul National University College of Medicine, Seoul, 03080 Republic of Korea; 2 Department of Agricultural Biotechnology, College of Agriculture and Life Science, Seoul National University, Seoul, 08826 Republic of Korea; 3 Institute of Endemic Diseases, Seoul National University College of Medicine, Seoul, 03080 Republic of Korea

**Keywords:** Bed bug, Multiplex PCR, Loop-mediated isothermal amplification, Molecular identification, *C. lectularius*, *C. hemipterus*

## Abstract

**Background:**

The resurgence of two bed bug species, the common bed bug (*Cimex lectularius* Linnaeus, 1758) and tropical bed bug (*Cimex hemipterus* Fabricius, 1803), in the same geographical regions has been frequently reported recently. Consequently, the rapid identification of these species is crucial for implementing targeted capture traps and tailored pyrethroid resistance diagnosis, due to differences in genetic and physiological traits.

**Methods:**

To develop molecular diagnostic methods, distinct protocols were established for multiplex PCR and loop-mediated isothermal amplification (LAMP) using species-specific primers based on species-specific segments of internal transcribed spacer 2 sequences. These methods were optimized for rapid and accurate identification of the two bed bug species.

**Results:**

Both multiplex PCR and LAMP protocols were effective in simultaneously identifying the two bed bug species, even when utilizing DNA released from dead specimens. Notably, the straightforward procedure and minimal time commitment of LAMP suggest its potential for rapid and accurate diagnosis of bed bugs in the field. The diagnostic accuracy of these methods was validated through a blind test.

**Conclusions:**

The multiplex PCR and LAMP protocols lay the foundation for rapid and accurate field identification of bed bug species, enabling the use of appropriate traps and the detection of species-specific pyrethroid resistance mutations. This approach ensures effective management tailored to the unique characteristics of each bed bug species.

**Graphical Abstract:**

**Figure:**
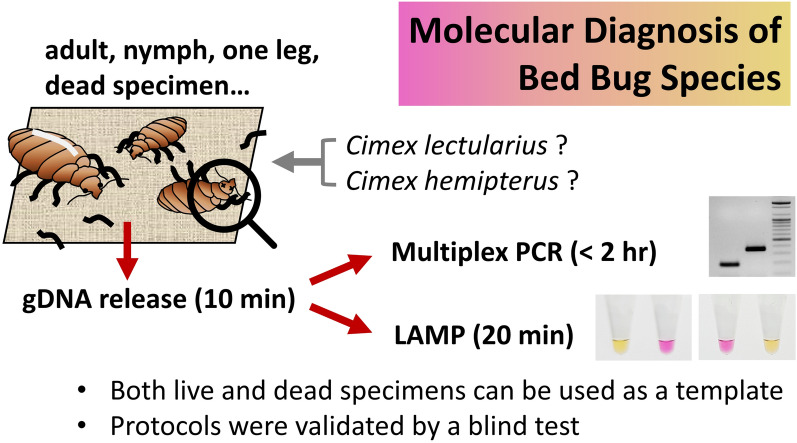

**Supplementary Information:**

The online version contains supplementary material available at 10.1186/s13071-024-06447-7.

## Background

The resurgence of two closely related bed bug species, the common bed bug (*Cimex lectularius* Linnaeus, 1758) and the tropical bed bug (*Cimex hemipterus* Fabricius, 1803) has become a global challenge over the past two decades [1]. This phenomenon is attributed to an increase in international travel and trade, coupled with the emergence of insecticide resistance [2–4]. Historically, *C. lectularius* has been prevalent in temperate zones, whereas *C. hemipterus* has predominantly been found in tropical and subtropical regions [5]. However, recent reports have indicated *C. hemipterus* occurrences in non-tropical regions, such as Russia [6], France [7], Italy [8], China [9], and central Europe [10] and *C. lectularius* infestations occur in tropical areas, such as Costa Rica [11]. A recent survey in the Republic of Korea (ROK) highlighted the predominant infestation of *C. hemipterus* (78.8%), replacing the previously endemic *C. lectularius* [12], challenging the notion of distinct climatic or environmental barriers to the distribution of this species.

Both *C. lectularius* and *C. hemipterus* exhibit nearly identical nesting and foraging behaviors as well as morphological characteristics [1]. Despite these similarities, unique differences in genetic and physiological traits necessitate tailored attention for each species. For instance, variations in voltage-sensitive sodium channel mutations (*C. lectularius* [13–15] and *C. hemipterus* [16, 17]) between two species underscore the importance of accurate species identification before detecting pyrethroid resistance mutations. Furthermore, the superior climbing ability of *C. hemipterus* emphasizes the need for rapid species identification to implement appropriate bed bug traps, as adult *C. hemipterus* can escape from smooth surface pitfall traps, unlike *C. lectularius* [18]. Therefore, conventional pitfall traps designed for *C. lectularius* would be ineffective for detecting, monitoring, or serving as a barrier against *C. hemipterus* [18]. In the case of a *C. hemipterus* infestation, only sticky traps would provide better outcomes for monitoring density and acting as a barrier. With this in mind, immediate identification of bed bug species during infestation is crucial for implementing targeted bed bug traps and tailored pyrethroid resistance diagnosis, especially in regions, such as the ROK, that face dual infestations of both *C. lectularius* and *C. hemipterus.* Consequently, there is an urgent need to develop rapid and accurate species identification tools that can be deployed in the field. Traditional identification methods rely on morphological characteristics, specifically the width-to-length ratio of the pronotum [5]. This method requires extensive examiner training and is only applicable to the adult stage, making it ineffective for immature stages. Although DNA barcoding utilizing the mitochondrial cytochrome oxidase subunit I (*mtCOI*) sequence as a marker can individually distinguish both species, it involves nucleotide sequencing, which requires at least one day [19, 20]. A multiplex PCR protocol using the mitochondrial *16S* rRNA sequence as a marker was developed to identify *C. lectularius* in eggs, leg fragments, and degraded samples [21]. However, no such system is currently available for identifying *C. hemipterus*.

This study aimed to establish molecular diagnostic protocols capable of simultaneously and accurately distinguishing between *C. lectularius* and *C. hemipterus* within a shorter time frame (30 min to 2.5 h). This efficiency makes the developed protocols suitable for the onsite diagnosis of bed bug species.

## Methods

### Bed bug strains and genomic DNA (gDNA) preparation

Two laboratory-maintained *C. lectularius* strains (FL, deltamethrin-susceptible; PT, deltamethrin-resistant) and one *C. hemipterus* strain (YS) were used for protocol development. Bed bugs were maintained using an artificial feeding system [13], which was reviewed and approved by the Institutional Review Board of Seoul National University (IRB No. E2211/001–003).

gDNA was extracted using two methods. First, the DNeasy Blood & Tissue Kit (Qiagen, Hilden, Germany) was used to extract gDNA from five adult bed bugs for protocol development. Briefly, female bed bugs were snap-frozen and homogenized in liquid nitrogen. Then, 180 μL of ATL buffer and 20 μL Proteinase K were added and incubated at 56 °C for 2 h. gDNA was purified using the manufacturer’s spin-column protocol. Alternatively, for gDNA release, a single adult, one 3rd instar nymph, or one leg detached from an adult was placed in 100, 30, or 10 μL of ddH_2_O, respectively, and then incubated at 95 ℃ for 10 min. The resulting supernatant was used as the template for gDNA amplification. For DNA release from the entire body of an adult or nymph, the abdomen was horizontally cut in half before soaking in ddH_2_O to enhance effective release.

### Sequencing of internal transcribed spacer 2 (ITS2) regions

To confirm the ITS2 sequences before protocol development, universal forward and two species-specific reverse primers were designed (Table 1). PCR products were generated from the extracted gDNA and each reaction mixture (20 μL) contained 5 ng of gDNA, 1.6 μL of 2.5 mM dNTPs, 2 μL of 10X polymerase buffer, 1 μL of each forward and reverse primer (5 μM), 0.4 μL of 50X Advantage 2 Polymerase Mix (Takara Biotechnology, Japan). PCR amplification was carried out in a T100™ Thermal Cycler (Bio-Rad, Hercules, CA, USA) and the reaction condition was as follows: 5 min preincubation at 95 °C; 35 cycles at 95 °C for 30 s, 62 °C for 20 s, 68 °C for 1 min; and final extension at 68 °C for 5 min. The PCR products were purified using a GeneAll^®^ Expin^™^ PCR SV (GeneAll Biotechnology, Korea) and sequenced (Macrogen, Seoul, Korea). The obtained sequences were compared with *ITS2* sequences of bed bugs from various countries (17 *C. lectularius* sequences from six countries and five *C. hemipterus* sequences from three countries). All primers used in this study were designed based on these identical regions, which were consistent among sequences from the same species across different geographical regions.

**Table 1:** Primers used in this study

Purpose	Species		Sequence (5’-3’)	amplicon
ITS2 amplification	Universal	F	CCTGTCTGAGGGTCGTTTTA	
	*Cimex lectularius*	R	TTCCAAGACGGTCAATAGGC	938 bp
	*Cimex hemipterus*	R	GATCTGAGGTCGAGTGTGTC	945 bp
Multiplex PCR	Universal	F	CCTGTCTGAGGGTCGTTTTA	
	*Cimex lectularius*	R	CCGCTGTTTCTACTTGTCCA	188 bp
	*Cimex hemipterus*	R	GACGTAACTCCGGTCTGGAT	362 bp
LAMP	*Cimex lectularius*	F3	CTCACAGTGCCTGGACCTA	
		B3	GTCTCAGTGGCAAACCCG	
		FIP	GGCAGCCTAACCGCTGTCTCTGCCTGTCTTTTGCTTGTCT	
		BIP	TCGGCATTTCCAGACCCGGAAGCTGAACGTCTGAAACGAC	
		LF	CTGGAAGCCGCATTGGGT	
		LB	CTCGAAGACGAGCACGATGG	
	*Cimex hemipterus*	F3	ATCCAGACCGGAGTTACGT	
		B3	GCTCGTCTCACACCAACAG	
		FIP	AAGGAAACCCGGAGCTCAACGACTGCCGTCCCGAGGA	
		BIP	CTAGCGCGTGCATCGCGCTGAAAAGCCGTCTGGGGC	
		LF	CCGAAACGACGCCTCCTTAC	
		LB	CACAGTGCCGTACCTATGCC	

### Species diagnosis using multiplex PCR

For multiplex PCR, a universal forward and two species-specific reverse primers were designed from the ITS2 sequences of *C. lectularius* and *C. hemipterus* to generate PCR products of different lengths. The reaction mixture (10 μL) contained template gDNA (1 ng of extracted gDNA or 1 μL of released gDNA), 0.8 µL of dNTPs (2.5 mM each), 1 μL of 10X buffer, 0.8 μL of forward primer (5 μM), 1.2 μL of reverse primer mix (species-specific primers of *C. lectularius* and *C. hemipterus*; 5 μM each), and 0.05 μL of Ex Taq (Takara). PCR was performed in T100^™^ Thermal Cycler (Bio-Rad) under the following conditions: 5 min preincubation at 95 °C; 35 cycles at 95 °C for 30 s, 64 °C for 30 s, and 72 °C for 1 min; and final extension at 72 °C for 5 min. Each product was visualized using electrophoresis on a 1.8% (w/v) agarose gel.

### Species diagnosis using loop-mediated isothermal amplification (LAMP)

For the LAMP reaction, F3, B3, FIP, BIP, LF, and LB primers were designed from species-specific regions in ITS2 sequences using the NEB LAMP Primer Design Tool (https://lamp.neb.com) (Table 1). LAMP was performed using WarmStart^®^ Colorimetric LAMP 2X Master Mix with UDG (NEB, Ipswich, MA, USA) according to the manufacturer’s protocol in a 10 μL reaction mixture with 1 ng of extracted gDNA or 1 μL of released gDNA as a template. Cross-reactivity between the two species was examined by replacing the template with the gDNA of the non-target bed bug species. Negative control reactions were performed without the template gDNA. The optimal reaction conditions were determined by investigating the reaction rates and false-positive detection across a range of temperatures (63, 65, and 67 °C), reaction times (20, 30, 40, and 50 min), and primer combinations comprising core (F3, B3, FIP, and BIP) and loop (LF and LB) primers using 1 ng of extracted gDNA.

### Blind test

The performance of the multiplex PCR-based diagnosis was validated using a blind test with 25 live or dead bed bug specimens, including adults and nymphs, randomly collected from laboratory colonies or field-collected populations (Additional file 1, Table S1). gDNA from severed whole bodies or legs of bed bugs was released in ddH_2_O following the protocol described above and analyzed using both multiplex PCR and the LAMP protocol by a graduate student not involved in this experiment. For LAMP reaction, results were observed after 20 min of incubation at 67 °C. To ensure the objectivity of the test, bed bug DNA specimens were prepared in one laboratory, and subsequent species diagnosis was conducted in another laboratory without any exchange of information regarding the specimens.

## Results

### Sequencing of ITS2 regions and design of primers for species diagnosis

**Fig. 1:**
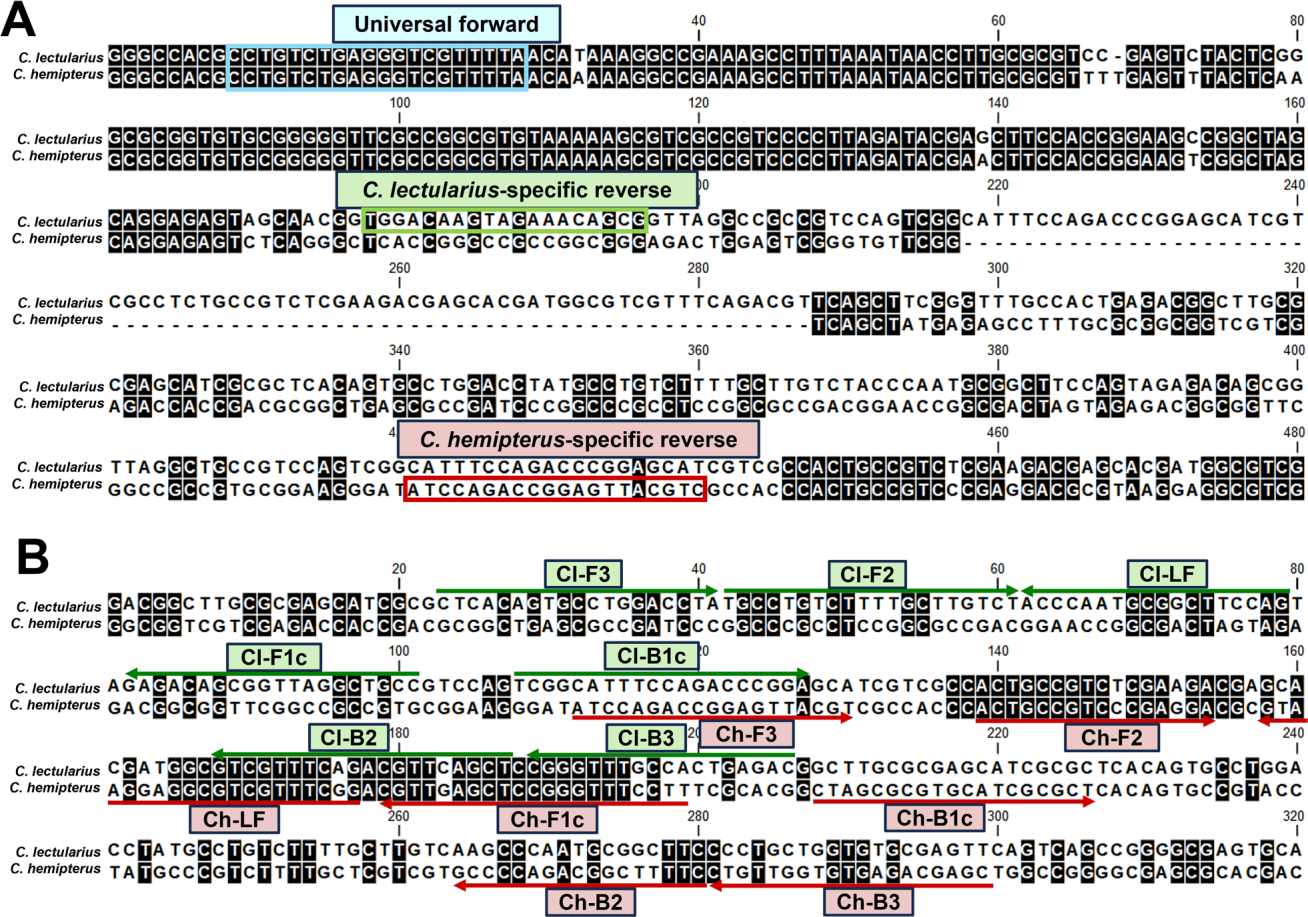
Primers for bed bug species identification. A segment of the internal transcribed spacer 2 (ITS2) sequences displaying the positions of (**A**) the multiplex PCR primers and (**B**) Loop-mediated isothermal amplification (LAMP) primers. Cl, *C. lectularius*; Ch, *C. hemipterus*

Partial ITS2 sequences of 938 and 945 bp were obtained from *C. lectularius* and *C. hemipterus*, respectively. Aligning these ITS2 sequences revealed a segment of identical sequences, from which a universal primer was derived for multiplex PCR analysis. Sequence variable regions were identified and used to design species-specific primers for multiplex PCR (Fig. 1A). The locations of the LAMP primers used for species diagnosis were illustrated in Fig. 1B. All LAMP primers were designed with a minimum nucleotide difference of 16.2% between *C. lectularius* and *C. hemipterus*. All primer sequences used for multiplex PCR and LAMP are listed in Table 1.

### Species diagnosis by multiplex PCR

**Fig. 2:**
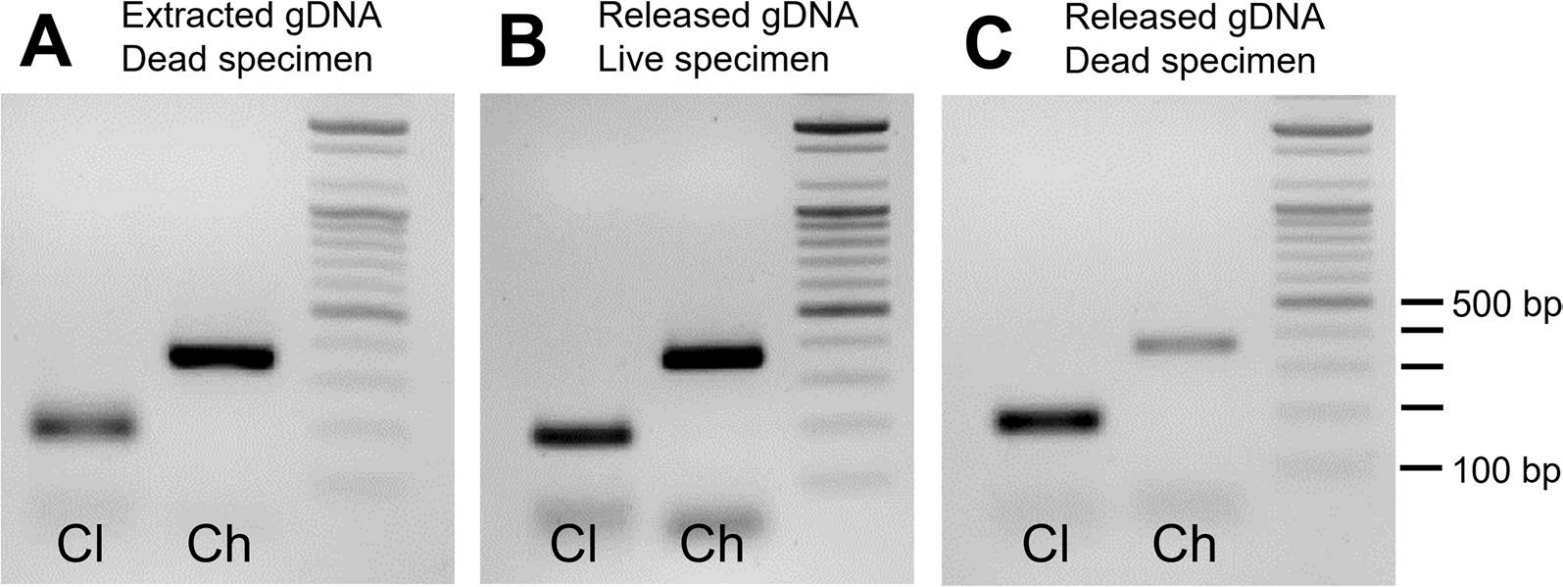
Results of multiplex conventional PCR using extracted and released DNA from bed bug specimens. Multiplex PCR was performed using templates composed of (**A**) extracted DNA from dead specimens, (**B**) released DNA from live specimens, and (**C**) released DNA from dead specimens. PCR products were visualized on a 1.8% agarose gel. Cl and Ch indicate the multiplex PCR bands specific to *C. lectularius* and *C. hemipterus,* respectively

Species-specific primer sets utilized in the multiplex PCR accurately identified the target bed bug species (Fig. 2). The *C. lectularius*-specific primer set produced a 188 bp PCR product, whereas the *C. hemipterus*-specific primer set resulted in a 362 bp PCR product. PCR amplification exhibited high species specificity, and importantly, the species-specific amplification of PCR products remained consistent regardless of the method or state of template DNA preparation. PCR amplification using DNA released from dead specimens was as robust as amplification using DNA extracted from dead or live specimens. This finding emphasizes the reliability of DNA released from dead bed bug specimens for multiplex PCR-mediated species diagnosis. The total time required for multiplex PCR-based species identification was approximately 2 h and 25 min, including the time for DNA release (10 min), PCR (1 h and 45 min), and agarose gel electrophoresis (30 min).

### Species diagnosis using LAMP

When utilizing the target-specific primer set, LAMP successfully generated a yellow color in positive reactions, facilitating precise identification of the target bed bug species. Optimal LAMP reaction conditions were determined by evaluating various temperature, primer sets and incubation times (Additional file 2, Fig. S1). The fastest detection of a positive result occurred when the reaction was conducted at 67 °C, using the same reaction time and primer sets. Among the various primer conditions tested, the reaction exhibited the fastest performance when both LF and LB primers were employed. However, a false-positive reaction (color change to yellow in the reaction without template gDNA) began to be observed 40 min after initiation of the reaction. Consequently, the optimal reaction conditions for further experiments were set to 67 °C with a 20 min incubation time.

**Fig. 3:**
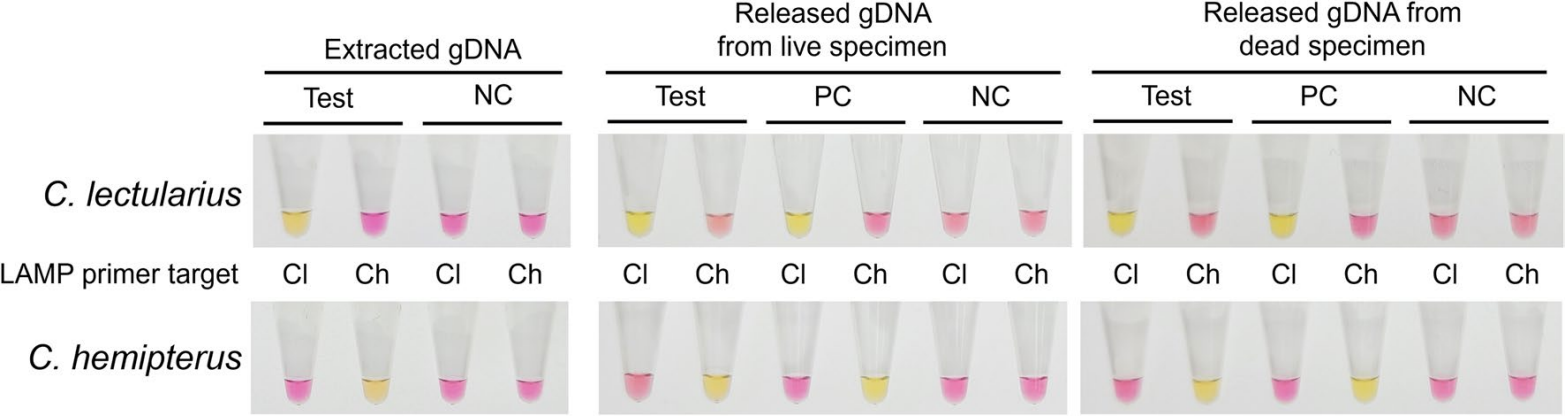
Results of loop-mediated isothermal amplification (LAMP) obtained after a 30-min reaction period. The yellow color in LAMP indicates a positive reaction for the respective target species, while the pink color indicates a negative reaction. Cl and Ch denote primers specific to *C. lectularius* and *C. hemipterus,* respectively. NC and PC indicate the negative control used to detect false positive reactions and the positive control containing extracted gDNA as a template, respectively

The quality and specificity of the LAMP reaction were unaffected by the method or state of template DNA preparation (Fig. 3). Similar to the multiplex PCR findings, the DNA released from dead specimens exhibited the capability generated a highly specific LAMP product, which was comparable to the results obtained using either extracted or released DNA from live specimens. Notably, no discernible cross-reactivity was observed between the two bed bug species, confirming the high accuracy of LAMP for species identification. All the reactions were confirmed by electrophoresis on a 1.8% (w/v) agarose gel (Additional file 3, Fig. S2). Including 10 min of DNA release, the total duration of LAMP-based species identification across multiple samples was approximately 30 min.

### Blind test for bed bug species diagnosis

**Fig. 4:**
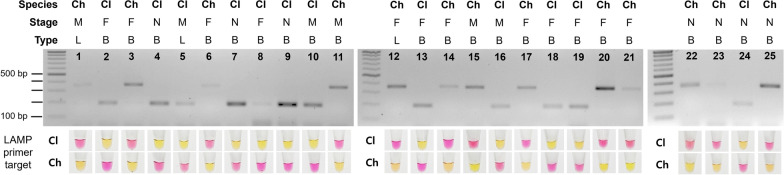
Blind test conducted using released DNA templates from 25 samples. Each sample was divided into two parts, with one part utilized for multiplex PCR and the other for loop-mediated isothermal amplification (LAMP). In LAMP, the yellow color indicates a positive reaction for the respective target species, while a pink color signifies a negative reaction. Cl, *C. lectularius*; Ch, *C. hemipterus*; M, male adult; F, female adult; N, nymph; L, leg; B, whole body

In a blind test involving 25 samples derived from live or dead specimens of two bed bug species at different developmental stages, prepared via gDNA extraction or release, species identification using either multiplex PCR or LAMP achieved 100% accuracy (Fig. 4; Additional file 4, Fig. S3). All 12 *C. lectularius* and 13 *C. hemipterus* specimens were correctly identified, irrespective of the diagnostic method used. Nymph specimens (specimens No. 4, 7, 9, 22, 23, 24, and 25) were accurately diagnosed, mirroring the precision observed in adult specimens (all specimens except No. 4, 7, 9, 22, 23, 24, and 25). Notably, no differences in the identification accuracy were found between live and dead specimens, and even one leg detached from an adult (specimens No. 1, 5, and 12) was suitable for this diagnostic method. These findings underscore the accuracy and reliability of both multiplex PCR and LAMP methods in diagnosing bed bug species across various specimen types, including both live and dead specimens, as well as specimens in the adult and nymphal stages.

## Discussion

In this study, two distinct protocols were developed for the simultaneous molecular identification of two bed bug species. Both multiplex PCR and LAMP successfully identified *C. lectularius* and *C. hemipterus* with high accuracy, as demonstrated in a blinded test. Traditional molecular identification based on *mtCOI* sequencing can accurately identify each bed bug species [19, 20], but is unsuitable for field applications because of the involvement of nucleotide sequencing. Although MALDI-TOF mass spectrometry has been employed to identify laboratory and wild-type strains of *C. lectularius* and *C. hemipterus* [22], it requires expensive laboratory instrumentation and is not suitable for field detection. While our study did not directly test the field adaptability of LAMP protocol, its simplified procedures and portable equipment suggest it has potential to facilitate immediate diagnosis of target bed bug species at infestation sites. Future studies should include field testing to validate these applications.

In the case of multiplex PCR, concurrent diagnosis was achieved in a single-tube reaction, thereby conserving reagents. However, this method requires an additional agarose gel electrophoresis step for species identification, which limits its utility for field diagnosis. To further improve the efficiency of the multiplex PCR protocol, the use of a genetically modified polymerase with a short extension time (e.g., ~ 5 s/kb), coupled with an elongation accelerator, could be considered. This approach significantly reduces the overall reaction time, making the PCR process faster and more suitable for rapid diagnosis in field conditions.

Conversely, LAMP, although requiring two separate tubes for species identification, can be conducted on-site using any portable, simple, and inexpensive thermal block for diagnosis owing to its isothermal amplification reaction. Its short reaction time, high sensitivity, and ease of scoring by the naked eye make it a suitable choice for on-site detection and point-of-care diagnosis. Despite these advantages, PCR is generally considered more robust than LAMP because of its potential for nonspecific amplification products under certain conditions, such as extended reaction times, non-optimized reaction temperatures, non-specific primer binding, and primer-dimer formation. Therefore, the choice between the two methods should consider the specific experimental purposes, laboratory settings, and available resources.

The ITS2 region of nuclear ribosomal DNA is considered a potential DNA barcode because of its conserved regions for universal primer design, easy amplification, and sufficient variability to distinguish closely related species [23]. The substantial variation in the ITS2 sequences between *C. lectularius* and *C. hemipterus* facilitated the design of species-specific primers for both multiplex PCR and LAMP. This primer design enables robust molecular diagnosis that can simultaneously identify both bed bug species.

Template DNA prepared using the DNA release method was sufficient for use in both the multiplex PCR and LAMP assays. The diagnostic resolution of the released DNA is comparable to that of the extracted DNA, leading to a substantial reduction in the time required for template DNA preparation. The straightforward procedure for DNA release involves heating severed specimens in ddH_2_O at 95 °C, facilitating the on-site preparation of template DNA for diagnostic purposes. While the alkaline DNA release buffer with EDTA for direct PCR [24] can be an alternative to ddH_2_O when long-term storage of released gDNA is necessary, it is only recommended for multiplex PCR. It is not suitable for the colorimetric LAMP method employed in this study due to unclear visual detection caused by the high pH of the buffer. However, considering that the production of protons and the subsequent drop in pH resulting from polymerase activity lead to a color change from pink to yellow in colorimetric LAMP, the use of an alkaline DNA release buffer is acceptable for conventional LAMP reactions where results are assessed using SYBR Green fluorescence dye (Additional file 5, Fig. S4).

DNA release from uncut adult specimens, regardless of whether they were live or dead, led to inconsistent diagnostic results, likely owing to the inefficiency of DNA extraction from intact bodies (data not shown). Severing the abdomen of bed bug specimens proved effective in obtaining the necessary amount of DNA required for downstream diagnosis, whether through multiplex PCR or LAMP. Given that the average DNA amount from a single severed adult bed bug specimen was 400–600 μg, the current DNA release protocol provides an ample DNA template for both quantitative and qualitative downstream diagnosis. As the current diagnostic protocol can be adapted as a bed bug detection tool, it is worthwhile investigating whether the current DNA release method can be applied to prepare DNA from bed bug feces, swabbed samples, or dust collected from potentially infested sites.

## Conclusions

Multiplex PCR and LAMP protocols were developed to facilitate the rapid and accurate diagnosis of the two bed bug species. Although multiplex PCR offers robust identification in laboratory settings, it may not be suitable for field diagnoses owing to its complexity. Conversely, LAMP has the potential for on-site detection and point-of-care diagnosis, due to its short reaction time, high sensitivity, enhanced readability, and low technique-dependency, although the field adaptability of LAMP protocol should be tested. Depending on the specific experimental requirements, laboratory conditions, and resource availability, either protocol should enable the use of appropriate traps and the timely detection of species-specific pyrethroid resistance mutations tailored to the unique characteristics of each bed bug species. Furthermore, the adaptability of similar protocols for identifying other medically significant pests, particularly during their immature stages, where morphological distinctions are challenging, offers promise for enhancing pest management strategies. By leveraging these protocols, efficient pest control measures can be implemented to improve public health and mitigate the spread of infestations.

## Supplementary Information

Additional file 1. Table S1.docx: Bed bug samples for the blind test.

Additional file 2. Fig. S1.tif: Optimization of LAMP reaction conditions for bed bug species identification. Temperatures, primer sets, and incubation times were evaluated for the LAMP assay. The yellow color indicates a positive reaction for the respective target species, while the pink color indicates a negative reaction. Cl and Ch denote the templates or primers specific to *C. lectularius* and *C. hemipterus*, respectively. In every fifth and sixth reaction, no gDNA template was included as a negative control to detect false-positive reactions.

Additional file 3. Figure S2. tif: Validation of LAMP reaction specificity in Figure 3 by gel electrophoresis. Only positive LAMP reactions showed distinct bands, confirming accurate amplification of target DNA. The absence of bands in negative controls indicates no nonspecific amplification, demonstrating the high specificity of the LAMP primers for identifying the species.

Additional file 4. Figure S3. tif: Validation of LAMP reaction specificity in Figure 4 (a blind test) by gel electrophoresis.

Additional file 5. Figure S4. tif: Evaluation of alkaline gDNA release buffer in LAMP reactions. LAMP reactions were conducted using template gDNA released in an alkaline gDNA release buffer or ddH_2_O. The results were evaluated based on (a) color change and (b) SYBR Green fluorescence dye. A positive reaction was indicated by a bright yellow color in both cases. When colorimetric LAMP was performed, the alkaline buffer affected the color change. The amplification results were validated by gel electrophoresis. NC indicates the negative control.

## Data Availability

The data supporting the findings of the study must be available within the article.

## Abbreviations

gDNA: Genomic DNA
LAMP: Loop-mediated isothermal amplification
*mtCOI*: Mitochondrial cytochrome oxidase subunit I
ROK: Republic of Korea
