# Supplementary material for: Development of molecular diagnostic protocols for simultaneous identification of common bed bugs (Cimex lectularius) and tropical bed bugs (Cimex hemipterus)
Source: Parasit Vectors. 2024 Oct 14;17:430. doi: 10.1186/s13071-024-06447-7 (PMC11476074; doi:10.1186/s13071-024-06447-7)
Supplement: Supplementary file 1 — Additional file 1. Table S1.docx: Bed bug samples for the blind test. [file 13071_2024_6447_MOESM1_ESM.docx]

**Table S1.** Bed bug samples for the blind test.

| **No.** | **Species** | **Developmental stage** | **Body part** | **Strain** |
| --- | --- | --- | --- | --- |
| 1 | *C. hemipterus* | Male adult | Legs | YS |
| 2 | *C. lectularius* | Female adult | Whole body | FL |
| 3 | *C. hemipterus* | Female adult | Whole body | YS |
| 4 | *C. lectularius* | 5^th^ instar nymph | Whole body | FL |
| 5 | *C. lectularius* | Male adult | Legs | PT |
| 6 | *C. hemipterus* | Female adult | Whole body | Field-collected |
| 7 | *C. lectularius* | 5^th^ instar nymph | Whole body | PT |
| 8 | *C. lectularius* | Female adult | Whole body | FL |
| 9 | *C. lectularius* | 5^th^ instar nymph | Whole body | FL |
| 10 | *C. lectularius* | Male adult | Whole body | FL |
| 11 | *C. hemipterus* | Male adult | Whole body | YS |
| 12 | *C. hemipterus* | Female adult | Legs | YS |
| 13 | *C. lectularius* | Female adult | Whole body | FL |
| 14 | *C. hemipterus* | Female adult | Whole body | Field-collected |
| 15 | *C. hemipterus* | Male adult | Whole body | YS |
| 16 | *C. lectularius* | Male adult | Whole body | FL |
| 17 | *C. hemipterus* | Female adult | Whole body | YS |
| 18 | *C. lectularius* | Female adult | Whole body | PT |
| 19 | *C. lectularius* | Female adult | Whole body | PT |
| 20 | *C. hemipterus* | Female adult | Whole body | Field-collected |
| 21 | *C. hemipterus* | Female adult | Whole body | YS |
| 22 | *C. hemipterus* | 5^th^ instar nymph | Whole body | YS |
| 23 | *C. hemipterus* | 5^th^ instar nymph | Whole body | Field-collected |
| 24 | *C. lectularius* | 5^th^ instar nymph | Whole body | FL |
| 25 | *C. hemipterus* | 5^th^ instar nymph | Whole body | YS |
