# Supplementary figures and images for: Development of molecular diagnostic protocols for simultaneous identification of common bed bugs (Cimex lectularius) and tropical bed bugs (Cimex hemipterus)
Source: Parasit Vectors. 2024 Oct 14;17:430. doi: 10.1186/s13071-024-06447-7 (PMC11476074; doi:10.1186/s13071-024-06447-7)

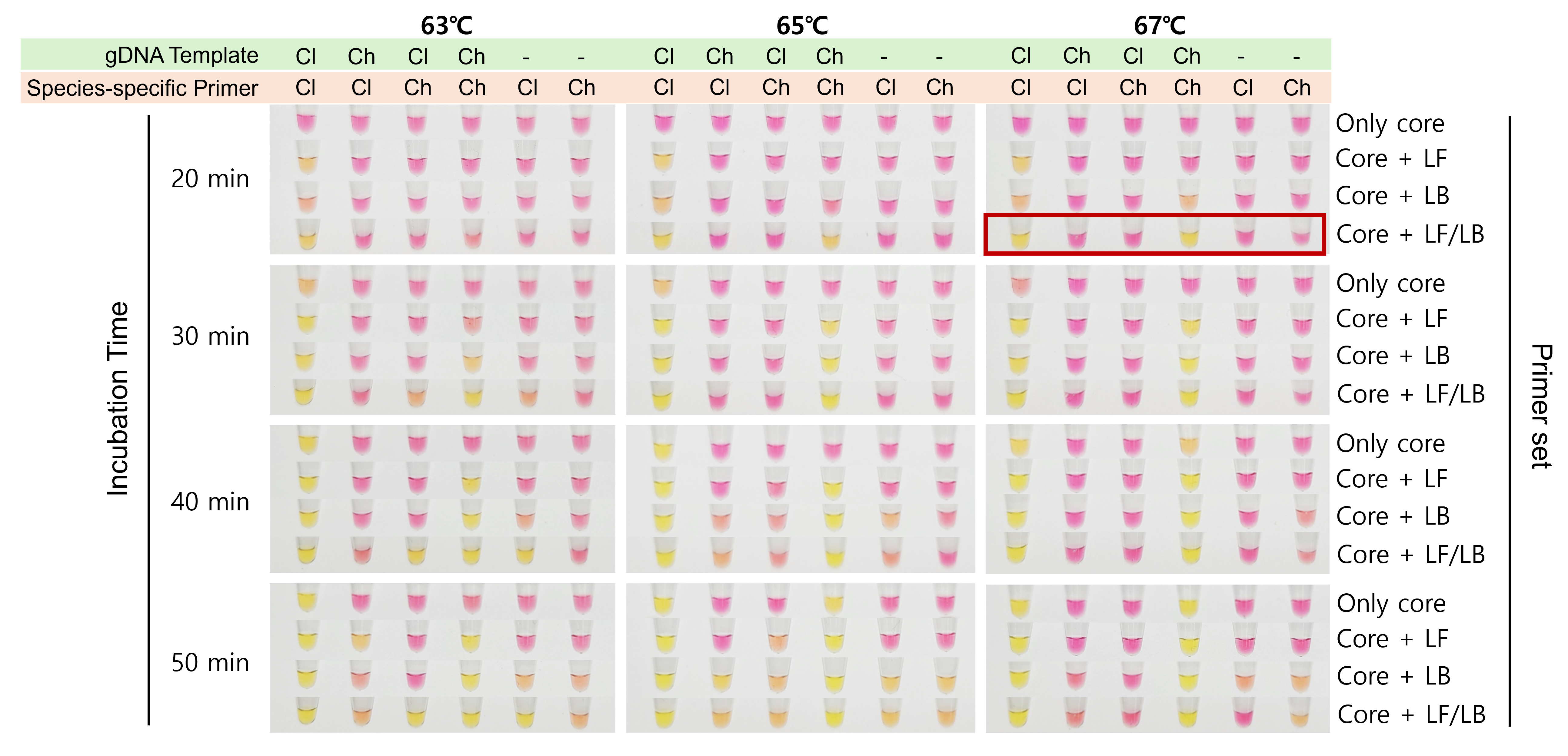

Supplement: Supplementary file 2 — Additional file 2. Fig. S1.tif: Optimization of LAMP reaction conditions for bed bug species identification. Temperatures, primer sets, and incubation times were evaluated for the LAMP assay. The yellow color indicates a positive reaction for the respective target species, while the pink color indicates a negative reaction. Cl and Ch denote the templates or primers specific to C. lectularius and C. hemipterus, respectively. In every fifth and sixth reaction, no gDNA template was included as a negative control to detect false-positive reactions. [file 13071_2024_6447_MOESM2_ESM.tif]

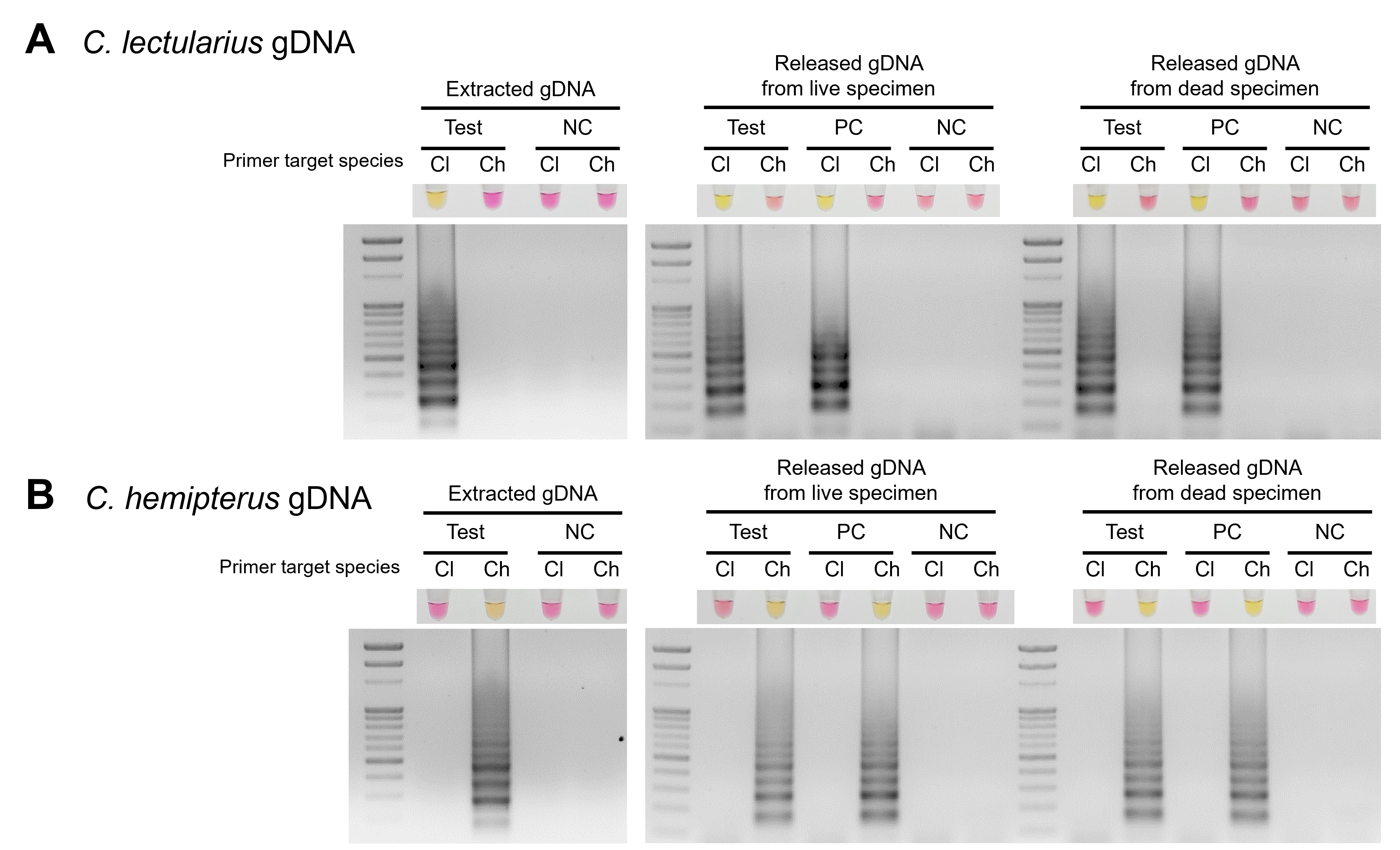

Supplement: Supplementary file 3 — Additional file 3. Figure S2. tif: Validation of LAMP reaction specificity in Figure 3 by gel electrophoresis. Only positive LAMP reactions showed distinct bands, confirming accurate amplification of target DNA. The absence of bands in negative controls indicates no nonspecific amplification, demonstrating the high specificity of the LAMP primers for identifying the species. [file 13071_2024_6447_MOESM3_ESM.tif]

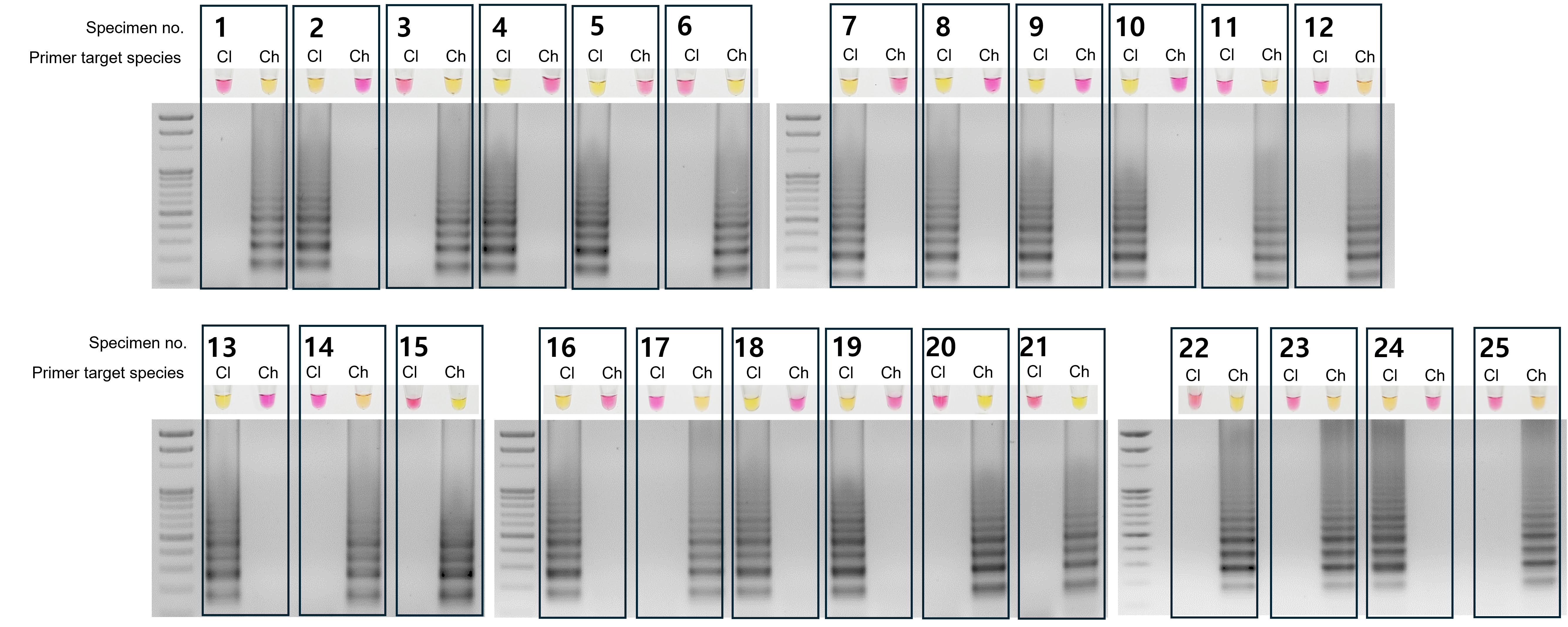

Supplement: Supplementary file 4 — Additional file 4. Figure S3. tif: Validation of LAMP reaction specificity in Figure 4 (a blind test) by gel electrophoresis. [file 13071_2024_6447_MOESM4_ESM.tif]

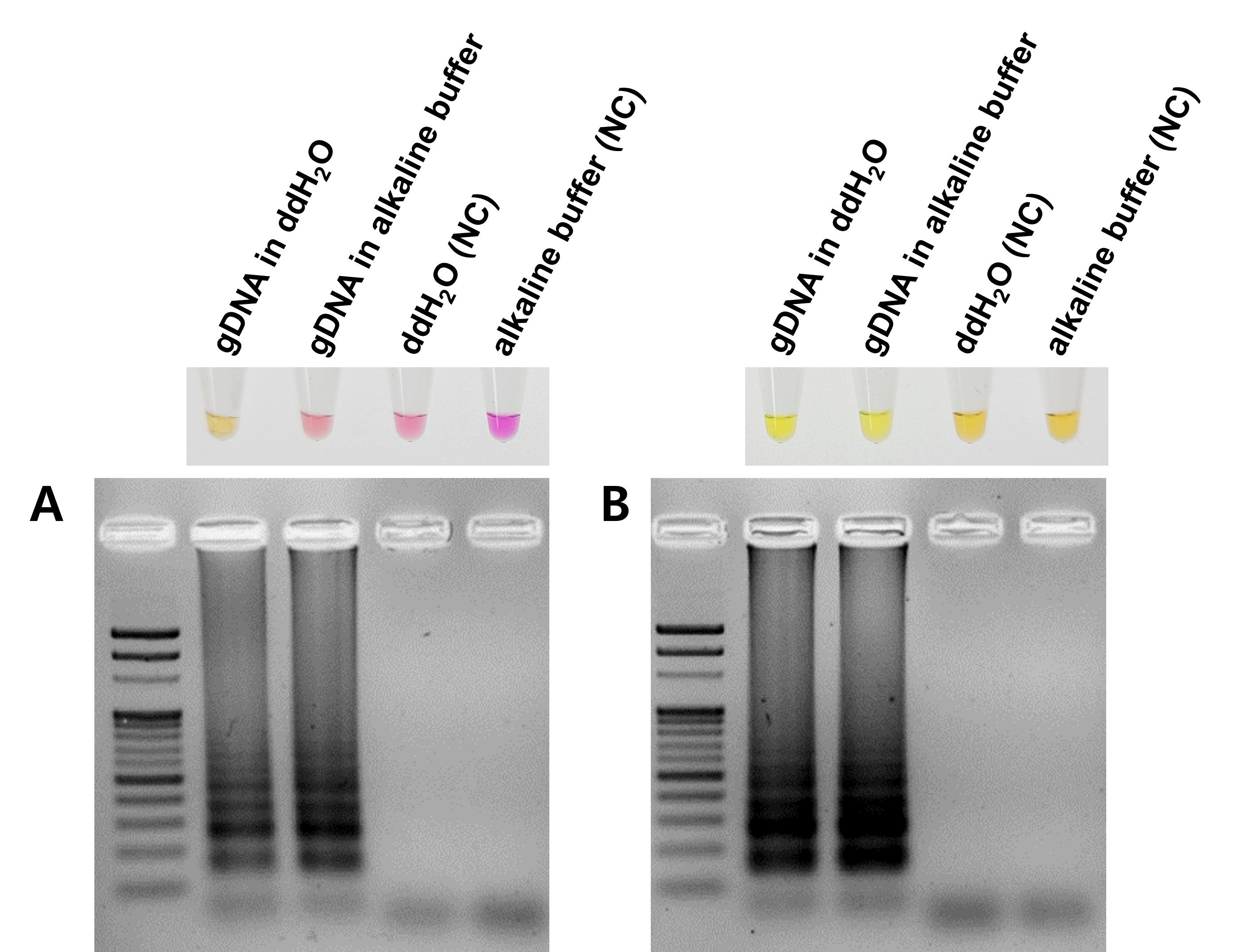

Supplement: Supplementary file 5 — Additional file 5. Figure S4. tif: Evaluation of alkaline gDNA release buffer in LAMP reactions. LAMP reactions were conducted using template gDNA released in an alkaline gDNA release buffer or ddH2O. The results were evaluated based on (a) color change and (b) SYBR Green fluorescence dye. A positive reaction was indicated by a bright yellow color in both cases. When colorimetric LAMP was performed, the alkaline buffer affected the color change. The amplification results were validated by gel electrophoresis. NC indicates the negative control. [file 13071_2024_6447_MOESM5_ESM.tif]
